# Serum Branched-Chain Amino Acid Metabolites Increase in Males When Aerobic Exercise Is Initiated with Low Muscle Glycogen

**DOI:** 10.3390/metabo11120828

**Published:** 2021-11-30

**Authors:** Lee M. Margolis, J Philip Karl, Marques A. Wilson, Julie L. Coleman, Claire C. Whitney, Stefan M. Pasiakos

**Affiliations:** 1 U.S. Army Research Institute of Environmental Medicine, Natick, MA 01760, USA; james.p.karl.civ@mail.mil (J.P.K.); marques.wilson.civ@mail.mil (M.A.W.); julie.l.coleman5.ctr@mail.mil (J.L.C.); claire.c.whitney.civ@mail.mil (C.C.W.); stefan.m.pasiakos.civ@mail.mil (S.M.P.); 2 Oak Ridge Institute of Science and Education, Oak Ridge, TN 37830, USA

**Keywords:** leucine, isoleucine, valine, protein breakdown

## Abstract

This study used global metabolomics to identify metabolic factors that might contribute to muscle anabolic resistance, which develops when aerobic exercise is initiated with low muscle glycogen using global metabolomics. Eleven men completed this randomized, crossover study, completing two cycle ergometry glycogen depletion trials, followed by 24 h of isocaloric refeeding to elicit low (LOW; 1.5 g/kg carbohydrate, 3.0 g/kg fat) or adequate (AD; 6.0 g/kg carbohydrate 1.0 g/kg fat) glycogen. Participants then performed 80 min of cycling (64 ± 3% VO_2_ peak) while ingesting 146 g carbohydrate. Serum was collected before glycogen depletion under resting and fasted conditions (BASELINE), and before (PRE) and after (POST) exercise. Changes in metabolite profiles were calculated by subtracting BASELINE from PRE and POST within LOW and AD. There were greater increases (*p* < 0.05, *Q* < 0.10) in 64% of branched-chain amino acids (BCAA) metabolites and 69% of acyl-carnitine metabolites in LOW compared to AD. Urea and 3-methylhistidine had greater increases (*p* < 0.05, *Q* < 0.10) in LOW compared to AD. Changes in metabolomics profiles indicate a greater reliance on BCAA catabolism for substrate oxidation when exercise is initiated with low glycogen stores. These findings provide a mechanistic explanation for anabolic resistance associated with low muscle glycogen, and suggest that exogenous BCAA requirements to optimize muscle recovery are likely greater than current recommendations.

## 1. Introduction

Performing aerobic exercise with low muscle glycogen stores has become popular over recent years [1,2,3,4]. Reducing muscle glycogen through sustained aerobic exercise followed by low-carbohydrate, high-fat feeding decreases the reliance on endogenous carbohydrate oxidation, while increasing fat oxidation during subsequent exercise bouts [2,3,5,6,7,8]. This practice facilitates training adaptations promoting use of fat as an energy source [9]. However, performing aerobic exercise with low glycogen stores may also compromise muscle repair and recovery [10,11] since the low muscle glycogen levels appear to increase the reliance on endogenous protein oxidation to meet metabolic demands [10,12], particularly branched-chain amino acids (BCAA). This results in lower rates of muscle protein synthesis [10,11] and blunts activation of the mechanistic target of rapamycin complex 1 (mTORC1) signaling pathway [13,14]. Furthermore, we recently reported reductions in the gene expression of myogenic markers when aerobic exercise was initiated with low versus adequate glycogen stores [15]. While these data indicate a potential reduction in the anabolic response to aerobic exercise when muscle glycogen content is low, the specific mechanism suppressing the anabolic state is unclear.

Untargeted metabolomics analysis can provide an insight into the metabolic mechanism underlying reduced anabolic responses to aerobic exercise when muscle glycogen is low [16,17]. Metabolomics analysis is an effective, sensitive, and comprehensive method for detecting changes in whole-body metabolism during acute or sustained exercise compared to resting/fasted conditions [18,19]. The most common and robust shift in metabolomics profiles following aerobic exercise is associated with fatty acid metabolism [18,19]; however, several investigations highlighted changes in amino acid metabolites that may indicate an increased reliance on BCAA for oxidative purposes [20,21,22]. We recently reported increased glycogenolysis during aerobic exercise under acute hypoxic exposure, resulting in greater decreases in leucine, isoleucine, and valine, and increases in downstream BCAA metabolites, compared to exercise in normoxic conditions [23]. Changes in BCAA metabolites, as well as increases in tricarboxylic acid (TCA) metabolites indicated the increased reliance of BCAA carbon skeletons for energy production through the TCA cycle with greater declines in glycogen stores during aerobic exercise [23]. However, whether the change in metabolomics profiles is the result of reductions in muscle glycogen or a response to aerobic exercise is unknown. To the best of our knowledge, no study has isolated the effect of glycogen content on metabolomics at the start of exercise.

The objective of this study was to assess the effects of initiating aerobic exercise with low or adequate muscle glycogen content on changes in global metabolomics profiles. We hypothesized that initiating aerobic exercise with a low glycogen content would result in large changes in fatty acid metabolites indicative of increased fat oxidation compared to adequate glycogen. Additionally, we hypothesized that a low glycogen content would produce an increase in branched-chain amino acid metabolites, indicating an increased breakdown of leucine, isoleucine, and valine, compared to adequate glycogen.

## 2. Results

### 2.1. Muscle Glycogen and Substrate Oxidation

As previously reported [9], muscle glycogen was manipulated by having participants complete a bout of glycogen depletion cycling at various work intensities until failure. Participants were then fed an isocaloric diet for the remainder of the day to elicit low (LOW) or adequate (AD) glycogen stores (Table 1). Muscle biopsies of the vastus lateralis were taken at baseline (BL) and after glycogen depletion (GD) to measure glycogen content. There was no difference between LOW and AD in glycogen at BL (LOW; 467 ± 95, AD; 472 ± 109 µmol/kg muscle dry weight) or GD (LOW; 207 ± 99, AD; 210 ± 145 µmol/kg muscle dry weight).

Participants returned to the laboratory the following day after a 10 h overnight fast. A pre-exercise (PRE) muscle biopsy was taken to determine glycogen content. Glycogen was lower (*p* < 0.05) in LOW (217 ± 103 µmol/kg muscle dry weight) compared to AD (396 ± 70 µmol/kg muscle dry weight). Immediately before starting the exercise bout, participants consumed 55 g of carbohydrate. Participants then began cycling for 80 min at their target VO_2_ (LOW; 65 ± 4, AD; 64 ± 3% VO_2_ peak) and consumed 30 g of carbohydrate at 20, 40, and 60 min during exercise. During steady-state cycling, carbohydrate oxidation was lower (*p* < 0.05) in LOW (1.59 ± 0.40 g/min) compared to AD (2.03 ± 0.36 g/min), while fat oxidation was higher (*p* < 0.05) in LOW (0.55 ± 0.10 g/min) compared to AD (0.38 ± 0.13 g/min). Immediately after (POST) exercise a final muscle biopsy was taken. Glycogen remained lower (*p* < 0.05) in LOW (137 ± 131 µmol/kg muscle dry weight) compared to AD (229 ± 94 µmol/kg muscle dry weight). Glycogen was lower (*p* < 0.05) at POST compared to PRE for AD, while there was no difference between PRE and POST in LOW.

### 2.2. Serum Metabolites

Serum samples for metabolomics analysis were collected by antecubital venipuncture before glycogen depletion under resting, fasted conditions (BASELINE), and before (PRE) and immediately after (POST) steady-state cycle ergometry, while consuming carbohydrate with LOW or AD glycogen. Metabolite profiles were analyzed in serum as change (Δ), calculated by subtracting the peak area from serum collected at BASELINE before glycogen depletion, from peak area serum collected at PRE and POST steady-state cycle ergometry within LOW and AD.

Metabolomics analysis detected 1329 metabolites. Of these, 1073 could be identified. In total, 309 metabolites demonstrated significant (*p* < 0.05, *Q* < 0.10) differences between treatment (LOW and AD) or time-by-treatment interactions. Of the identified significant metabolites, 22% were within amino acid metabolism pathways, and 56% were within fatty acid metabolism pathways.

**Figure 1:**
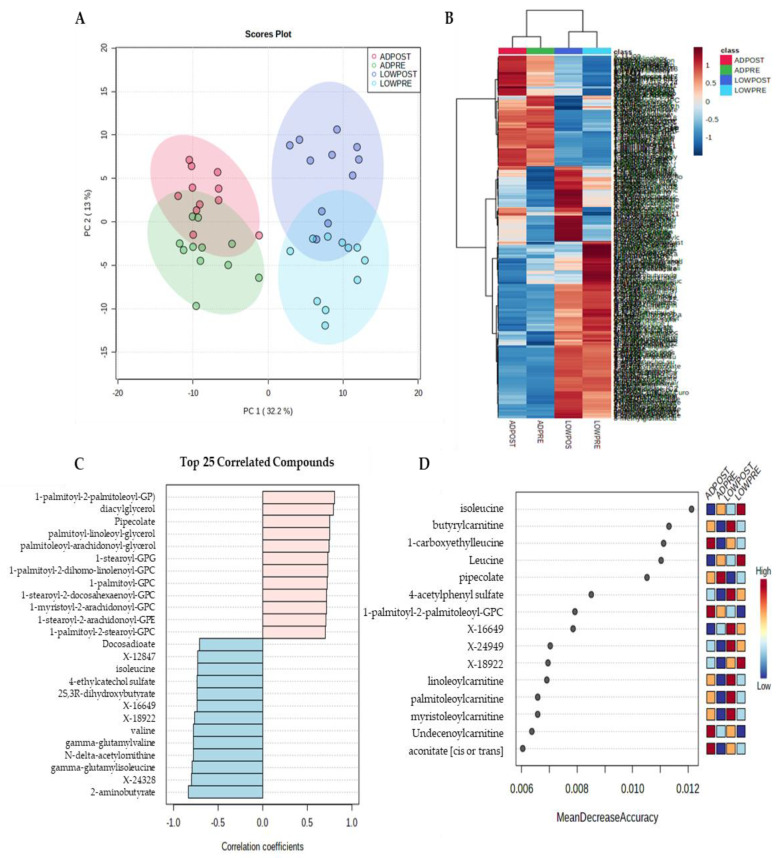
Principal component (**A**), hierarchical clustering (**B**), pattern hunter (**C**) and random forest plot (**D**) analysis; *n* = 11.

Principal components analysis and hierarchical clustering of Euclidean distances show a clear separation in metabolite profiles by treatment (Figure 1A,B). Pattern search using a Pearson correlation coefficient and random forest plot analysis identified multiple metabolites that were most strongly associated between treatments and the best-predicted treatment and time point assignment (Figure 1C,D). Metabolites identified in a pattern search and random forest plot analysis were primarily related to fatty acid and BCAA metabolism. Given that fatty acid and BCAA metabolites were identified using non-targeted, unbiased analysis, remaining statistical analysis focused on these pathways.

**Figure 2:**
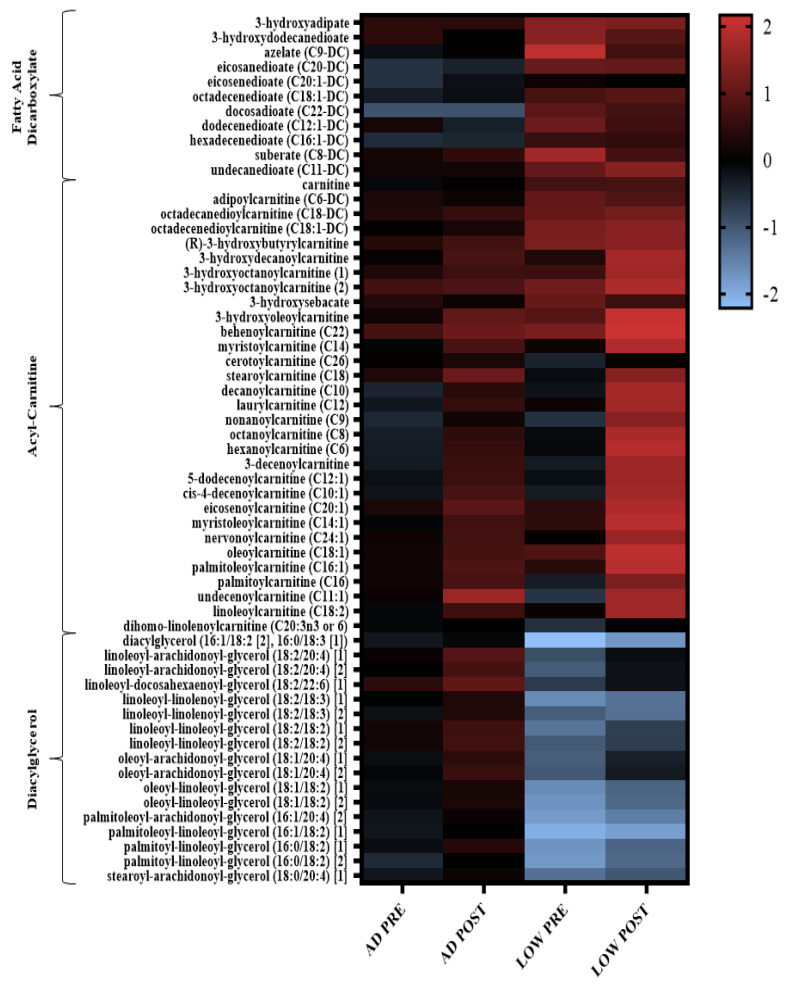
Heatmap of the change in diglycerol, acyl-carnitine, and fatty acid dicarboxylate metabolites that had a significant main effect of treatment (LOW compared to AD) or time-by-treatment interactions; *n* = 11, *p* < 0.05, *Q* < 0.10. Mean ± SD data with *p* and *Q* values reported in Supplemental Table S1.

In agreement with the increased fat oxidation measured by indirect calorimetry reported in the parent paper [9], when exercise was initiated with LOW versus AD glycogen content, there were robust changes in fatty acid metabolites, indicating increased fatty acid mobilization and oxidation PRE and POST aerobic exercise (Figure 2, Supplemental Material, Table S1). Specifically, 80% of diacylglycerol metabolites had a greater decrease (*p* < 0.05, *Q* < 0.10) in both PRE and POST during LOW compared to AD. The main effect of treatment and time-by-treatment interactions were observed for the change in acyl-carnitine metabolites, with 69% of these metabolites having greater increases, PRE and/or POST (*p* < 0.05, *Q* < 0.10), in LOW compared to AD (Supplemental Table S1). The main effects of treatment and time-by-treatment interactions were observed in the change in fatty acid dicarboxylate, with 37% of these metabolites having greater increases, PRE and/or POST (*p* < 0.05, *Q* < 0.10), in LOW compared to AD (Supplemental Table S1). Distinctions between treatment and the time-by-treatment interactions of individual metabolites within these sub-pathways is located in Supplemental Table S1.

**Figure 3:**
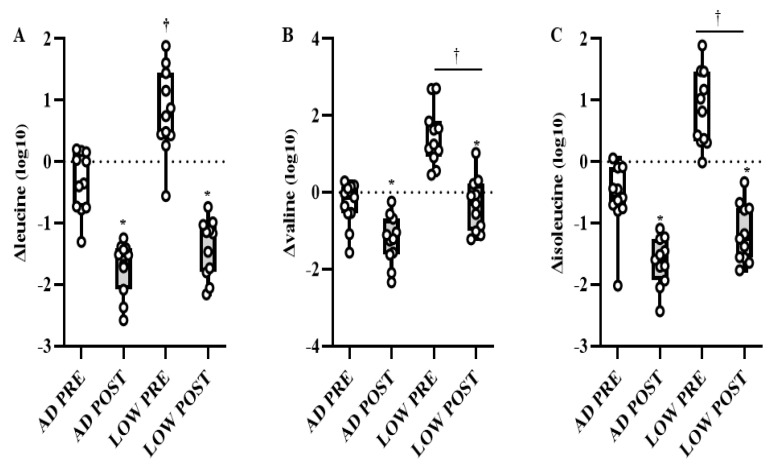
Mean ± SD of change in leucine (**A**), valine (**B**), and isolecuine (**C**) from BASELINE to PRE and POST aerobic exercise following 24 h of refeeding to elicit adequate (AD) or low (LOW) glycogen stores. *n* = 11, * Different from PRE; *p* < 0.05, *Q* < 0.10. † Different from AD; *p* < 0.05, *Q* < 0.10.

**Figure 4:**
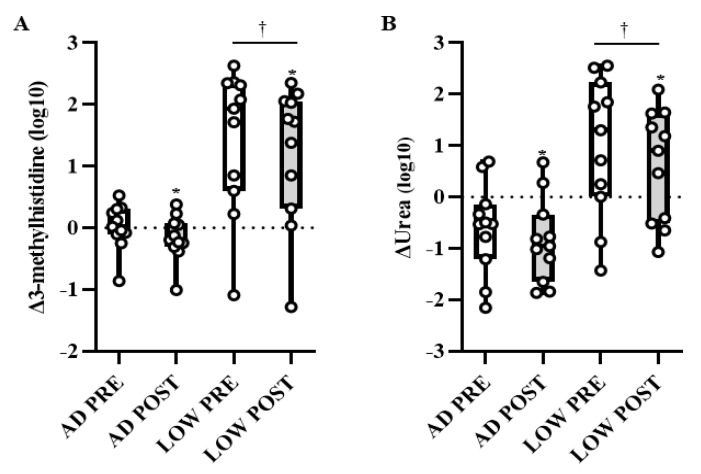
Mean ± SD of change in urea (**A**) and 3-methylhistidine (**B**) from BASELINE to PRE and POST aerobic exercise following 24 h of refeeding to elicit adequate (AD) or low (LOW) glycogen stores. *n* = 11, * Different from PRE; *p* < 0.05, *Q* < 0.10. † Different from AD; *p* < 0.05, *Q* < 0.10.

There was a time-by-treatment interaction in the BCAA leucine, with PRE being higher (*p* < 0.05, *Q* < 0.10) in LOW compared to AD (Figure 3A). Independent of time, valine and isoleucine were higher (*p* < 0.05, *Q* < 0.10) in LOW compared to AD (Figure 3B,C). Regardless of treatment, leucine, isoleucine, and valine were lower (*p* < 0.05, *Q* < 0.10) in POST compared to PRE (Figure 3A–C). Additionally, the main effects of treatment and time-by-treatment interactions were observed in downstream BCAA metabolites, with 64% of these metabolites having greater increases from BASELINE to PRE and/or POST (*p* < 0.05, *Q* < 0.10) in LOW compared to AD (Table 2). The change in markers of protein breakdown, urea and 3-methylhistidine, were higher (*p* < 0.05, *Q* < 0.10) in LOW compared to AD, independent of time (Figure 4A,B).

## 3. Discussion

The main finding from this study was that initiating aerobic exercise with low glycogen concentrations alters global metabolomics profiles before and after exercise compared to when exercise is initiated with adequate glycogen. Differences in metabolomics profiles between LOW and AD were within sub-pathways of fatty acid metabolism and amino acid metabolism. Greater increases in BCAA and acyl-carnitine metabolites, and urea and 3-methylhistidine, indicate there is an increased reliance on BCAA catabolism for substrate oxidation when aerobic exercise is initiated in LOW compared to AD muscle glycogen.

Low energy and/or glycogen availability increases the reliance of BCAA as substrates for energy production and the maintenance of glucose homeostasis [24,25]. In the current study, greater deceases in leucine, isoleucine, and valine, at POST compared to PRE, regardless of treatment, suggest that BCAA were used as an energy source during aerobic exercise regardless of glycogen content. Reductions in circulating BCAA are in agreement with previous studies assessing the change in circulating metabolites after aerobic exercise compared to resting fasted conditions [20,21,26,27]. However, though each BCAA decreased in POST compared to PRE aerobic exercise in the current study, downstream BCAA metabolites, such as 3-methyl-2-oxovalerate, 4-methyl-2-oxopentanoate, and 3-hydroxyisobutyrate, had greater increases in LOW compared to AD. Concomitant reductions in circulating BCAA, with increases in downstream BCAA, indicate an increased reliance on BCAA carbon skeletons for energy production in LOW compared to AD [28]. Our laboratory recently linked similar alterations in circulating BCAA metabolites, resulting from increased glycogenolysis and reductions in endogenous glucose stores to reduced mTORC1 activation in response to aerobic exercise during acute hypoxia exposure [23,29]. Additionally, in the current study, there was a greater increase in circulating urea and 3-methylhistidine in LOW compared to AD. Greater increases in these indirect markers of myofibrillar protein breakdown substantiates our hypothesis that exercising with low muscle glycogen accelerates muscle protein loss by increasing the body’s reliance on BCAA for energy production. As a result, BCAA, to support muscle protein synthesis and anabolic signaling during and in recovery from exercise is diminished when aerobic exercise is initiated with low glycogen [10,11,13,14,15].

**Figure 5:**
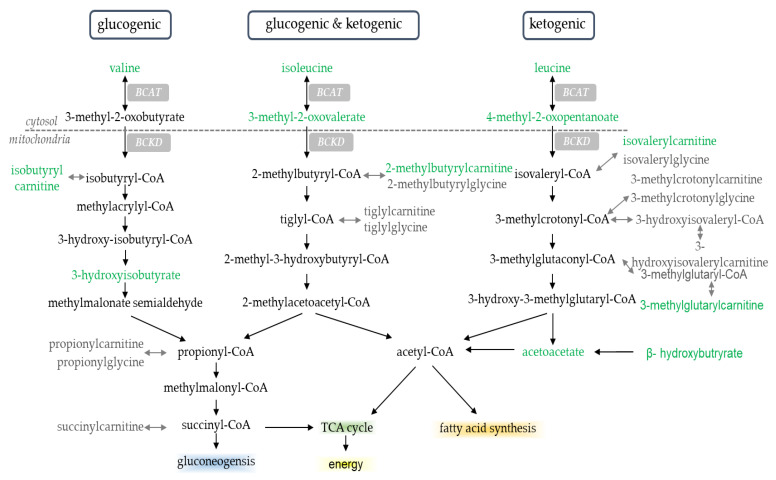
Branched-chain amino acid metabolites. Metabolites highlighted in green were significantly different in LOW compared to AD; *p* < 0.05, *Q* < 0.10. Endpoint of metabolism (e.g., TCA cycle, fatty acid synthesis, energy, and gluconeogenesis) highlighted for effect.

Changes in acyl-carnitine metabolites in the current study are consistent with results from the parent paper [9], reporting a ~2-fold increase in rates of whole-body fat oxidation and an increased transcriptional regulation of fatty acid transport and oxidation within skeletal muscle when participants initiated aerobic exercise with LOW compared to AD glycogen. Increases in acyl-carnitine metabolites are indicative of increased fatty acid oxidation [19,30,31,32,33]. These metabolites are formed within mitochondria when fatty acyl-CoAs are carnitized to acyl-carnitines by carnitine palmitoyltransferase 1 (CPT1) to allow for the shuttling of fatty acids from the cytosol of the cell into the mitochondria, where they can be used as fuel via β-oxidation [34]. We previously reported that within these same participants, muscle CPT1 gene expression increased, PRE and POST aerobic exercise, in LOW compared to AD [9]. Concurrent increases in these metabolites and their transcriptional regulators provide further evidence that changes in these metabolites are reflective of shifts in substrate oxidation at the tissue level. Increases in circulating acyl-carnitine metabolites may also be indicative of a metabolic shift from an anabolic to catabolic state [35]. There is overlap between BCAA and acyl-carnitine metabolites [36,37], with several acyl-carnitine metabolites, such as isobutyrylcarnitine, butyrylcarnitine, 2-methylbutyrylcarnitine, and 3-methylglutarylcarnitine, as downstream byproducts of BCAA breakdown (Figure 5). Greater increases in these metabolites in LOW compared to AD in the current study further suggest an increased reliance on BCAA of oxidative purposes when glycogen availability is limited. Alterations in use of BCAA oxidation with low glycogen availability likely contributed to reductions in markers of myogenesis, PAX7, MYOD, and MYOGENIN within the skeletal muscle of these same participants [15]. Indeed, low glucose availability in cell culture and mouse models was shown to increase BCAA catabolism and impair cellular growth [38]. Additionally, in cell culture models where lipid-enriched media are used to increase fatty acid metabolism through the increased expression of molecular regulators of fat oxidation, such as CPT1 and fat acid translocase (FAT), a reduced myotube formation occurs due to lower expression of myogenic regulator factor genes, including PAX7, MYOD, and MYOGENIN [39]. Ultimately, these data suggest that changes in substrate metabolism resulting from low glycogen availability may enhance the breakdown of BCAA, which can impair muscle growth and recovery follow exercise.

The increased reliance on endogenous BCAA for fuel use during aerobic exercise when glycogen stores are low, suggests an increased requirement of exogenous BCAA consumption to overcome the blunted anabolic response and optimize muscle recovery. While adequate availability of all amino acids is necessary for the synthesis of new muscle and whole-body proteins, BCAA availability is required to drive increases in protein synthesis [40,41,42]. Without adequate replenishment of the BCAA pool, protein breakdown will continue to exceed muscle protein synthesis resulting in a net negative protein balance [40,41,42]. To compensate for increased endogenous BCAA catabolism with low glycogen stores, greater amounts of exogenous BCAA must be consumed during or post-exercise for the adequate stimulation of protein synthesis, in order to maintain or increase net protein balance [43]. Indeed, recent work from our laboratory [44], reported that, following 5 days of a 30% energy deficit, consuming ~24 g essential amino acids (~13 g BCAA) resulted in a greater net positive protein balance compared to ~8 g essential amino acids (~4 g BCAA) post-exercise. Though glycogen was not measured in this previous work [44], given the severity and length of the deficit, it may be assumed the glycogen availability was low. Future investigation is warranted to determine if increased BCAA intake offsets the anabolic resistance associated with initiating aerobic exercise with low glycogen stores.

Greater decreases in diacylglycerol and increases in dicarboxylate fatty acid metabolites with LOW compared to AD are also consistent with the increased fat oxidation reported in the parent paper [9]. The shifts in fatty-acid-related metabolites were likely the result of reduced glycogen availability at the onset of aerobic exercise, as well as the 24 h of high fat (70% total kcal) refeeding after a bout of glycogen-depleting cycling. Past studies reported changes in metabolites post aerobic exercise that were predominately related to fatty acid metabolism [30,31,32,45,46,47,48]. Changes in fatty acid metabolites following prolonged aerobic exercise were linked to declines in muscle glycogen content post compared to pre exercise [30]. In the current study, decreases in diacylglycerol metabolites in LOW compared to AD likely suggest an increased muscle uptake is used as substrate for β-oxidation [49]. These results are in agreement with previous results from our laboratory [32], reporting reductions in diacylglycerol metabolites following 4 days of arduous military training, in which participants experienced a 55% energy deficit. Additionally, increases in dicarboxylate fatty acids in LOW compared to AD in the current study are in agreement with previous studies [21,32,48], reporting increased concentrations of these metabolites following sustained aerobic exercise. A higher concentration of dicarboxylate fatty acids reflects increases in fat oxidation though ω-oxidation, an alternative oxidative pathway to β-oxidation. While ω-oxidation typically accounts for a relatively small amount of fatty acid oxidation, increases in dicarboxylate fatty acids may indicate higher levels of ω-oxidation to compensate for saturation in β-oxidation [50].

Our current study provides a novel insight into the changes of metabolomics profiles when aerobic exercise is initiated with LOW compared to AD glycogen stores. However, this work is not without its limitations. This study was a secondary analysis from a larger investigation that was performed to detect differences in substrate oxidation using indirect calorimetry between treatments [9]. This sample size was appropriate to address the studies primary outcomes; however, it is relatively small for a metabolomics analysis. Though relatively small, the alterations in metabolomics profiles largely align with changes in the substrate oxidation when aerobic exercise was initiated with LOW or AD glycogen [9]. The agreement between indirect calorimetry and metabolomics datasets, along with larger differences in the effects between treatments, enhances the physiological relevance and likely validity of the results. Additionally, the use of a randomized crossover study design enhanced the statistical power of our work by allowing each participant to act as their own control. Furthermore, participants’ dietary intake and exercise was controlled 48 h prior to baseline measurements within each treatment, as well the 24 h post-glycogen depletion leading to the steady-state aerobic exercise protocol day. This level of control over exercise and diet allows for the further isolation of the effect of glycogen status on metabolomics profiles. It should also be noted that this work should be interpreted in the context of the participants that were studied, which were recreationally active males. While females were not excluded from participation in this study, only males consented to enroll in the study. The responses of metabolomics profiles to low glycogen availability may differ in females [51,52] and elite athletes [53]. Finally, the dietary protein intake of our controlled diet was statistically different between groups. However, though dietary protein intake was statistically significant, a difference of 0.1 g/kg is likely physiologically irrelevant and would not result in the large changes in BCAA metabolites observed in our current study.

In conclusion, results from this study indicate that when aerobic exercise is initiated with low glycogen, there are large differences in global metabolomics profiles before and after exercise compared to adequate glycogen stores. Differences in metabolomics profiles between treatments were primarily driven by changes in metabolites related to fatty acid and amino acid metabolism. Greater increases in BCAA and acyl-carnitine metabolites, as well as urea and 3-methylhistidine, suggest that when aerobic exercise is initiated low muscle glycogen, the reliance on BCAA catabolism for substrate oxidation is greater than when exercise is initiated with adequate glycogen stores. Findings may provide a metabolic explanation for why muscle is anabolically resistant to the mechanical stimulus of exercise, and suggest exogenous BCAA requirements to optimize muscle recovery are likely increased when glycogen stores are limited.

## 4. Materials and Methods

### 4.1. Participants

Twelve healthy, non-obese, recreationally active men between the ages of 18–39 years were enrolled to participate in this randomized, crossover study after providing informed, written consent. Participants in this study were part of a larger investigation that also aimed to assess the impact of glycogen content on the rates of exogenous glucose oxidation during steady-state aerobic exercise (www.clinicaltrials.gov, NCT03250234, 15 August 2017). The results from this separate objective are reported elsewhere [9]. Data are reported on 11 of the 12 enrolled participants due to insufficient serum from 1 participant. This study was approved by the Institutional Review Board at the US Army Medical Research and Development Command (MRDC, Fort Detrick, MD) and data collection took place at the US Army Research Institute of Environmental Medicine (USARIEM, Natick, MA, USA).

Height (Seritex, Inc., Carlstadt, NJ, USA), body mass (WB-110 A, Tanita, Tokyo, Japan), and body composition (dual energy x-ray absorptiometry, DPX-IQ, GE Lunar Corporation, Madison, WI, USA) were measured to characterize participants. Peak oxygen uptake (VO_2_ peak) was determined using a progressive-intensity cycle ergometer (Lode, BV, The Netherlands) test and an indirect, open-circuit respiratory system (Parvo TrueOne 2400, Parvomedics, Sandy, UT, USA). Exercise intensities for protocol days were based on volunteers’ VO_2_ peak. Volunteer characteristics were: 21 ± 4 years, 1.8 ± 0.1 m; 83 ± 11 kg body mass; 26 ± 2 kg/m^2^ body mass index; 19 ± 10 kg fat mass; 63 ± 9 kg fat-free mass; and 44 ± 4 mL/kg/min VO_2_ peak.

### 4.2. Experimental Design

To normalize glycogen content, 48 h prior to testing, participants completed a glycogen depletion protocol by cycling at various intensities until failure [28], followed by ingestion of a controlled diet to replete glycogen content (Supplemental Table S1). At the conclusion of the repletion phase, a baseline (BASELINE) muscle biopsy was obtained from the vastus lateralis under local anesthesia (lidocaine) after a 10 h overnight fast. Participants then completed the same glycogen depletion (GD) protocol, immediately followed by a muscle biopsy. Participants then consumed a controlled diet designed to elicit low (LOW) or adequate (AD) glycogen stores for 24-h. Participants returned to the laboratory the following morning after a 10 h overnight fast, and performed 80 min of steady-state cycle ergometry (64 ± 3% VO_2_ peak) while ingesting 146 g of carbohydrate (95 g glucose + 51 g fructose; 1.8 g/min). Muscle biopsies were obtained before (PRE) and after (POST) steady-state exercise to assess glycogen content. Following a minimum 7-d washout period, participants returned to the laboratory to complete the second arm of the investigation. Treatment (LOW vs. AD) order was randomized using a random numbers generator to avoid order bias.

### 4.3. Glycogen Depletion Protocol

Exercise intensities were based on peak oxygen uptake (VO_2_ peak) determined using a progressive-intensity cycle ergometer (Lode, BV, The Netherlands) test and an indirect, open circuit respiratory system (True Max 2400, Parvomedics, Sandy, UT, USA). To deplete muscle glycogen, participants completed 2 min of high-intensity cycling (work period) at 90% VO_2_ peak, followed by a 2 min recovery period cycling at 50% VO_2_ peak [54]. This work-to-recovery ratio was maintained until the participant was no longer able to complete 2 min of cycling at 90% VO_2_ peak. Cycling intensity during the work period was progressively lowered to 80%, 70%, and 60% VO_2_ peak when the participant was unable to complete 2 min of cycling at the given workload. Once the participant could not complete 2 min of cycling at 60% VO_2_ peak, exercise was stopped. The recovery period was maintained at 50% VO_2_ peak. Participants performed two practice sessions to ensure that they were familiar with the protocol before testing. Glycogen depletion exercise time (LOW: 84 ± 25, AD: 88 ± 24 min) and intensity (mean power; LOW: 164 ± 26, AD: 161 ± 25 watts) were similar between treatments [9].

### 4.4. Study Diets

Dietary intake was controlled for 48 h before testing began, to ensure participants presented for both trials with similar glycogen stores. Participants were all provided food and beverages (except water), consuming, on average, 5.7 ± 0.6 g/kg/d carbohydrate, 1.2 ± 0.1 g/kg/d protein, and 1.0 ± 0.1 g/kg/d fat in both study arms during the normalization phase. On glycogen depletion protocol days, participants consumed a high fat or high carbohydrate diet for 24 h to elicit LOW or AD glycogen stores at the start of steady-state exercise (Table 1). Study dietitians prepared and administered all meals, derived from Meal, Ready-to-Eat (MRE; Ameriqual, Evansville, IN, USA) combat rations and commercially available food items. Participants returned all wrappers to dietitians to confirm intake. Participants abstained from nicotine and alcohol use during the study.

### 4.5. Steady-State Cycle Ergometry

Participants ingested 146 g total carbohydrate (95 g glucose + 51 g fructose), dissolved in 1450 mL of water, at an average ingestion rate of 1.8 g/min. Immediately before starting the exercise bout, participants ingested 550 mL (56 g) of the carbohydrate drink. Participants then began cycling for 80 min at their target VO_2_ (64 ± 3% VO_2_ peak). Participants consumed 300 mL (30 g) of the carbohydrate drink at 20, 40, and 60 min during exercise. The carbohydrate drink was prepared by the Combat Feeding Directorate (Natick, MA, USA) contained corn-derived crystalline fructose (KRYSTAR^®^ 300, Tate and Lyle Sugars, London, UK), maltodextrin (MALTRIN QD^®^ M500, Grain Processing Corporation, Muscatine, IA, USA), and dextrose (CERELOSE^®^, Ingredion, Westchester, IL, USA). Nutrient content was confirmed before use with gas chromatography (Covance Laboratories, Inc., Madison, WI, USA).

### 4.6. Metabolomics

Serum samples for metabolomics analysis were collected by antecubital venipuncture before glycogen depletion under resting, fasted conditions (BASELINE), before (PRE) and after (POST) steady-state cycle ergometry while consuming carbohydrate at with LOW or AD glycogen. Samples were centrifuged at 3000 rpm at 4 °C for 10 min. Serum was then stored at −80 °C until analysis. Samples were analyzed using four separate methods: two separate reverse-phase (RP)/UPLC-MS/MS methods with positive ion mode electrospray ionization (ESI), a RP/UPLC-MS/MS method with negative ion mode ESI, and a HILIC/UPLCMS/MS method with negative ion mode ESI (Metabolon Inc., Morrisville, NC, USA). Technical replicates, blanks, internal standards, and several recovery standards were analyzed with experimental samples for quality control. Raw data were extracted, peaks identified, and quality control processed using proprietary hardware and software. The relative quantitation values are based on integrated peak areas (area under the curve). All samples were analyzed on an equivalency basis based on volume.

Metabolites were identified by automated comparison of the ion features in the experimental samples using a references library of chemical standard entries that included retention time, molecular weight (m/z), preferred adducts, and in-source fragments, as well as associated MS spectra, and were curated by visual inspection for quality control using software developed at Metabolon (Metabolon Inc., Morrisville, NC, USA) [55,56]. The level of identification for the majority of the compounds detected meets the highest standard of metabolite identification according to the Metabolomics Standards Initiative [57]. Several types of controls were analyzed in concert with the experimental samples. A pooled matrix sample was generated by taking a small volume of each experimental sample to serve as a technical replicate throughout the data set. Extracted water samples served as process blanks. A cocktail of quality control standards that would interfere with the measurement of endogenous compounds was spiked into every analyzed sample to allow instrument performance monitoring and aided chromatographic alignment. Instrument variability was determined by calculating the median relative standard deviation (RSD) for the standards that were added to each sample prior to injection into the mass spectrometers. Overall process variability was determined by calculating the median RSD for all endogenous metabolites (i.e., non-instrument standards) present in 100% of the pooled matrix samples.

### 4.7. Statistical Analysis

Analyses were completed using R v4.0.3, SPSS v26 (IBM Analytics; Armonk, NY, USA), ArrayStudio (Omicsoft Corp.; Cary, NC, USA), and MetaboAnalyst v.5.0 (https://www.metaboanalyst.ca) [58]. Before analysis of metabolomics data, any missing values were imputed using the minimum observed peak area for each compound. Peak areas for each metabolite were then normalized to set the mean equal to 0, and log10 transformed to meet model assumptions. Metabolite profiles were analyzed in serum as change (Δ), calculated by subtracting peak area from serum collected, PRE and POST steady-state cycle ergometry, from peak area baseline serum collected before glycogen depletion within LOW and AD.

Principal components analysis, hierarchical clustering of Euclidean distances, pattern hunter, and random forest plot analysis were conducted using MetaboAnalyst v.5.0 (45) to assess the effect of change from baseline at PRE and POST within and between LOW and AD on changes in global metabolite profiles. Mixed-model, repeated-measures ANOVA was used to determine main effects of change in time (PRE vs. POST), treatment (LOW vs. AD), and time-by-treatment interactions. To account for multiple comparisons, the Benjamini–Hochberg method was used to estimate false discovery rate (*Q*-value). Statistical significance was set at *p* < 0.05 and *Q* < 0.10. 

## Figures and Tables

**Table 1:** Energy and relative macronutrient intake.

	Low	Adequate	*p* Value
**Energy (kcal/d)**	3081 ± 374	3086 ± 347	0.77
**Carbohydrate (g/kg/d)**	1.5 ± 0.1	6.0 ± 0.2	<0.01
**Fat (g/kg/d)**	3.0 ± 0.5	1.0 ± 0.5	<0.01
**Protein (g/kg/d)**	1.3 ± 0.5	1.2 ± 0.5	<0.01

Mean ± SD, *n* = 11.

**Table 2:** Branched-chain amino acid metabolites.

	AD		LOW			*p*, *Q* Values	
Metabolite	PRE	POST	PRE	POST	Time	Treatment	T × T
**1-carboxyethylleucine**	−0.47 ± 0.31	1.59 ± 0.40	0.11 ± 0.77	1.60 ± 0.66	<0.01, <0.01	0.19, 0.20	0.01, 0.04
**2-hydroxy-3-methylvalerate**	−0.08 ± 0.31	0.13 ± 0.41	1.27 ± 0.61	1.00 ± 0.87	0.77, 0.79	<0.01, <0.01	0.03, 0.11
**3-methyl-2-oxovalerate**	−0.42 ± 0.65	−0.06 ± 0.59	0.59 ± 0.83	0.16 ± 0.82	0.70, 0.73	0.05, 0.06	<0.01, <0.01
**4-methyl-2-oxopentanoate**	−0.27 ± 0.70	0.18 ± 0.70	0.50 ± 1.05 ^†^	0.00 ± 1.04	0.81, 0.83	0.43, 0.43	<0.01, <0.01
**alpha-hydroxyisocaproate**	−0.17 ± 0.48	−0.32 ± 0.83	0.98 ± 0.74 ^†^	0.01 ± 1.00 *	<0.01, <0.01	0.03, 0.03	0.01, 0.04
**alpha-hydroxyisovalerate**	0.09 ± 0.51	0.26 ± 0.45	1.34 ± 0.57	1.30 ± 0.72	0.24, 0.29	<0.01, <0.01	0.06, 0.19
**beta-hydroxyisovalerate**	0.03 ± 0.41	0.08 ± 0.35	0.91 ± 0.50	0.87 ± 0.54	0.93, 0.94	<0.01, <0.01	0.26, 0.51
**ethylmalonate**	0.01 ± 0.29	0.46 ± 0.37	0.27 ± 0.22	1.01 ± 0.66	<0.01, <0.01	0.02, 0.03	0.07, 0.22
**N-acetylisoleucine**	−0.74 ± 0.54	−0.09 ± 0.71 *	0.88 ± 1.27 ^†^	0.35 ± 1.48	0.82, 0.84	0.01, 0.02	0.03, 0.11
**2-ketocaprylate**	−0.20 ± 0.37	0.06 ± 0.35	0.76 ± 0.68	1.26 ± 0.49	<0.01, <0.01	<0.01, <0.01	0.15, 0.35
**2-methylbutyrylcarnitine (C5)**	−0.28 ± 0.49	0.30 ± 0.63	0.69 ± 0.41	1.82 ± 0.62	<0.01, <0.01	<0.01, <0.01	0.02, 0.10
**3-hydroxyisobutyrate**	−0.06 ± 0.69	0.95 ± 0.69	0.88 ± 0.63	1.77 ± 0.78	<0.01, <0.01	<0.01, <0.01	0.74, 0.86
**3-methylglutaconate**	−0.04 ± 0.39	0.15 ± 0.46	0.67 ± 0.68	1.23 ± 0.65	<0.01, <0.01	<0.01, <0.01	0.05, 0.16
**3-methylglutarylcarnitine (2)**	0.12 ± 0.48	0.12 ± 0.60	−0.84 ± 0.40	−0.49 ± 0.45	0.12, 0.16	<0.01, <0.01	0.12, 0.30
**isobutyrylcarnitine (C4)**	−0.26 ± 0.50	0.18 ± 0.68	0.57 ± 0.88	1.59 ± 0.71	<0.01, <0.01	<0.01, <0.01	0.02, 0.10
**isovalerylcarnitine (C5)**	0.02 ± 1.16	0.73 ± 0.94	0.41 ± 0.53	1.99 ± 0.74 ^†^*	<0.01, <0.01	0.01, 0.02	0.06, 0.18
***n*-acetylleucine**	−0.83 ± 0.93	0.00 ± 0.91	0.36 ± 0.87	0.34 ± 1.19	0.02, 0.03	0.06, 0.07	0.01, 0.07
**butyrylcarnitine (C4)**	−0.27 ± 0.23	0.47 ± 0.28	−0.26 ± 0.18	1.04 ± 0.56 ^†^*	0.00, 0.00	0.02, 0.03	0.01, 0.04

Mean ± SD of log10 transformed change in branched-chain amino acid metabolites from BASELINE to PRE and POST aerobic exercise following 24 h of refeeding, in order to elicit adequate (AD) or low (LOW) glycogen stores. *n* = 11, * Indicates time-by-treatment interaction, where POST is different from PRE within a treatment; *p* < 0.05, *Q* < 0.10. ^†^ Indicates time-by-treatment interaction, where LOW is different from AD at a given time point; *p* < 0.05, *Q* < 0.10.

## Data Availability

The data presented in this study are available on request from the corresponding author. The data are not publicly available due to retention of data for future publications.

## Supplementary Materials

The following are available online at https://www.mdpi.com/article/10.3390/metabo11120828/s1, Table S1: Fatty acid metabolites.
