# Supplementary material for: Serum Branched-Chain Amino Acid Metabolites Increase in Males When Aerobic Exercise Is Initiated with Low Muscle Glycogen
Source: Metabolites. 2021 Nov 30;11(12):828. doi: 10.3390/metabo11120828 (PMC8708125; doi:10.3390/metabo11120828)
Supplement: Supplementary file 1 [file metabolites-11-00828-s001.zip › metabolites-1463906-supplementary.pdf]

Supplemental Table S1: Fatty Acid Metabolites

	AD		LOW			*P , Q* Values	
**Metabolite**	**PRE**	**POST**	**PRE**	**POST**	**Time**	**Treatment**	**T x T**
**Fatty Acid Dicarboxylate**							
3-hydroxyadipate	0.46 ± 0.39	0.43 ± 0.65	1.42 ± 0.61	1.29 ± 0.81	0.38 , 0.45	< 0.01 , 0.01	0.57 , 0.80
3-hydroxydodecanedioate	0.47 ± 0.56	-0.02 ± 0.30	1.43 ± 0.89	0.91 ± 0.93	< 0.01 , < 0.01	< 0.01 , 0.01	0.83 , 0.90
azelate (C9-DC)	-0.18 ± 0.78	0.03 ± 0.68	2.00 ± 1.22	0.67 ± 0.89*	< 0.01 , 0.01	< 0.01 , < 0.01	< 0.01 , < 0.01
eicosanedioate (C20-DC)	-0.58 ± 0.58	-0.39 ± 0.39	1.08 ± 0.86	1.06 ± 0.67	0.29 , 0.35	< 0.01 , < 0.01	0.16 , 0.37
eicosenedioate (C20:1-DC)	-0.58 ± 0.57	-0.19 ± 0.28	0.11 ± 0.74	0.00 ± 0.51	0.26 , 0.32	0.04 , 0.05	0.05 , 0.18
octadecenedioate (C18:1-DC)	-0.34 ± 0.43	-0.16 ± 0.57	0.75 ± 0.57	0.91 ± 0.66	0.08 , 0.11	< 0.01 , < 0.01	0.90 , 0.94
docosadioate (C22-DC)	-0.97 ± 0.55	-0.95 ± 0.68	0.96 ± 0.73	0.71 ± 1.08	0.41 , 0.48	< 0.01 , < 0.01	0.35 , 0.61
dodecenedioate (C12:1-DC)	0.22 ± 0.65	-0.37 ± 0.67	1.13 ± 0.95	0.64 ± 1.11	< 0.01 , < 0.01	0.01 , 0.02	0.65 , 0.82
hexadecenedioate (C16:1-DC)	-0.50 ± 0.29	-0.44 ± 0.41	0.57 ± 0.57	0.51 ± 0.70	1.00 , 1.00	< 0.01 , < 0.01	0.56 , 0.80
suberate (C8-DC)	0.21 ± 1.05	0.49 ± 0.71	1.70 ± 1.19†	0.69 ± 0.90*	0.04 , 0.06	0.04 , 0.05	< 0.01 , 0.01
undecanedioate (C11-DC)	0.19 ± 0.61	0.19 ± 0.48	1.04 ± 1.33	1.41 ± 1.29	0.16 , 0.21	0.02 , 0.03	0.16 , 0.36
2S,3R-dihydroxybutyrate	0.43 ± 0.58	0.10 ± 0.60	1.90 ± 0.78	1.59 ± 0.74	< 0.01 , < 0.01	< 0.01 , < 0.01	0.81 , 0.89
2-hydroxydecanoate	-0.80 ± 0.87	-0.89 ± 0.71	0.56 ± 0.82	1.00 ± 0.81	0.16 , 0.21	< 0.01 , < 0.01	0.03 , 0.13
2-hydroxyoctanoate	-0.46 ± 0.43	0.30 ± 0.43	0.50 ± 0.65	1.53 ± 0.64	< 0.01 , < 0.01	< 0.01 , < 0.01	0.07 , 0.21
3-hydroxydecanoate	0.37 ± 0.70	0.34 ± 0.47	0.96 ± 0.55	1.29 ± 0.91	0.25 , 0.30	0.01 , 0.02	0.17 , 0.37
3-hydroxyhexanoate	0.17 ± 0.67	0.53 ± 0.38	1.20 ± 0.65	1.44 ± 0.79	0.01 , 0.01	< 0.01 , < 0.01	0.57 , 0.80
3-hydroxylaurate	0.39 ± 0.77	0.58 ± 0.68	0.86 ± 0.58	1.46 ± 0.89	0.01 , 0.01	0.03 , 0.04	0.13 , 0.32
3-hydroxyoctanoate	0.00 ± 0.61	0.44 ± 0.43	0.83 ± 0.52	1.46 ± 0.71	< 0.01 , < 0.01	< 0.01 , < 0.01	0.36 , 0.61
5-hydroxyhexanoate	-0.29 ± 0.97	-0.14 ± 1.02	0.80 ± 0.70	0.89 ± 0.81	0.23 , 0.29	0.01 , 0.02	0.76 , 0.87
2-hydroxymyristate	0.14 ± 0.83	-0.09 ± 0.94	-0.26 ± 0.46	-1.25 ± 0.73†*	< 0.01 , < 0.01	0.01 , 0.02	0.02 , 0.08
**Acyl-Carnitine**							
adipoylcarnitine (C6-DC)	0.31 ± 0.56	0.13 ± 0.67	1.03 ± 0.66	0.83 ± 0.72	0.05 , 0.08	0.01 , 0.02	0.87 , 0.92
octadecanedioylcarnitine (C18-DC)	0.36 ± 0.68	0.54 ± 0.68	1.06 ± 0.28	1.22 ± 0.35	< 0.01 , < 0.01	0.01 , 0.01	0.74 , 0.86
octadecenedioylcarnitine (C18:1-DC)	0.04 ± 0.67	0.25 ± 0.69	1.26 ± 0.38	1.44 ± 0.40	< 0.01 , < 0.01	< 0.01 , < 0.01	0.75 , 0.87
(R)-3-hydroxybutyrylcarnitine	0.38 ± 0.67	0.68 ± 0.85	1.28 ± 0.73	1.44 ± 0.75	0.06 , 0.09	0.01 , 0.02	0.53 , 0.78
3-hydroxydecanoylcarnitine	0.09 ± 0.69	0.74 ± 0.75	0.33 ± 0.55	1.73 ± 0.57†	< 0.01 , < 0.01	0.03 , 0.03	< 0.01 , 0.01
3-hydroxyoctanoylcarnitine (1)	0.32 ± 0.85	0.64 ± 0.54	0.61 ± 0.73	1.69 ± 0.93†*	< 0.01 , < 0.01	0.04 , 0.05	0.01 , 0.06
3-hydroxyoctanoylcarnitine (2)	0.69 ± 0.74	0.79 ± 0.62	1.18 ± 0.66	1.82 ± 0.71	0.00 , 0.01	0.01 , 0.02	0.03 , 0.12
3-hydroxysebacate	0.37 ± 0.46	0.12 ± 0.36	1.07 ± 0.54	0.63 ± 0.70	< 0.01 , < 0.01	0.01 , 0.02	0.29 , 0.54
3-hydroxyoleoylcarnitine	0.16 ± 0.43	1.02 ± 0.53	0.90 ± 0.63	2.09 ± 0.68	< 0.01 , < 0.01	< 0.01 , < 0.01	0.03 , 0.11
behenoylcarnitine (C22)	0.71 ± 0.80	1.11 ± 0.76	1.27 ± 0.78	2.17 ± 0.59†	< 0.01 , < 0.01	0.02 , 0.02	< 0.01 , 0.01
myristoylcarnitine (C14)	-0.06 ± 0.46	0.76 ± 0.43	0.15 ± 0.46	1.85 ± 0.53†	< 0.01 , < 0.01	< 0.01 , 0.01	< 0.01 , < 0.01
cerotoylcarnitine (C26)	0.04 ± 0.40	0.27 ± 0.38	-0.41 ± 0.18	0.09 ± 0.37	< 0.01 , < 0.01	0.01 , 0.02	0.14 , 0.33
stearoylcarnitine (C18)	0.32 ± 0.60	1.11 ± 0.54	-0.13 ± 0.55	1.42 ± 0.65	< 0.01 , < 0.01	0.77 , 0.77	< 0.01 , < 0.01
decanoylcarnitine (C10)	-0.42 ± 0.65	0.44 ± 0.73	-0.23 ± 0.63	1.73 ± 0.60†	< 0.01 , < 0.01	0.01 , 0.02	< 0.01 , < 0.01
laurylcarnitine (C12)	-0.27 ± 0.58	0.56 ± 0.66	0.12 ± 0.43	1.70 ± 0.55†	< 0.01 , < 0.01	< 0.01, 0.01	< 0.01 , < 0.01
nonanoylcarnitine (C9)	-0.46 ± 0.66	0.17 ± 0.85	-0.56 ± 0.55	1.43 ± 0.61†	< 0.01 , < 0.01	0.03 , 0.04	< 0.01 , < 0.01
octanoylcarnitine (C8)	-0.36 ± 0.65	0.49 ± 0.70	-0.12 ± 0.59	1.80 ± 0.62†	< 0.01 , < 0.01	0.01 , 0.01	< 0.01 , < 0.01
hexanoylcarnitine (C6)	-0.31 ± 0.61	0.56 ± 0.56	-0.09 ± 0.47	1.90 ± 0.70†	< 0.01 , < 0.01	< 0.01 , 0.01	< 0.01, 0.01
3-decenoylcarnitine	-0.30 ± 0.70	0.60 ± 0.87	-0.30 ± 0.55	1.66 ± 0.55†	< 0.01 , < 0.01	0.06 , 0.06	< 0.01 , 0.01
5-dodecenoylcarnitine (C12:1)	-0.19 ± 0.66	0.63 ± 0.78	-0.17 ± 0.55	1.64 ± 0.64†	< 0.01 , < 0.01	0.06 , 0.07	< 0.01 , < 0.01
cis-4-decenoylcarnitine (C10:1)	-0.24 ± 0.61	0.75 ± 0.64	-0.31 ± 0.66	1.71 ± 0.67†	< 0.01 , < 0.01	0.10 , 0.10	< 0.01 , < 0.01
eicosenoylcarnitine (C20:1)	0.29 ± 0.64	0.94 ± 0.73	0.45 ± 0.68	1.85 ± 0.70†	< 0.01 , < 0.01	0.07 , 0.08	< 0.01 , 0.01
myristoleoylcarnitine (C14:1)	-0.06 ± 0.47	0.69 ± 0.53	0.48 ± 0.47	1.94 ± 0.63	< 0.01 , < 0.01	< 0.01 , < 0.01	< 0.01 , < 0.01
nervonoylcarnitine (C24:1)	0.14 ± 0.44	0.72 ± 0.53	0.07 ± 0.79	1.60 ± 0.83†	< 0.01 , < 0.01	0.09 , 0.10	0.01 , 0.06
oleoylcarnitine (C18:1)	0.19 ± 0.41	0.73 ± 0.42	0.82 ± 0.45	1.99 ± 0.64	< 0.01 , < 0.01	< 0.01 , < 0.01	< 0.01 , 0.02
palmitoleoylcarnitine (C16:1)	0.17 ± 0.44	0.82 ± 0.37	0.40 ± 0.49	1.94 ± 0.65†	< 0.01 , < 0.01	0.00 , 0.01	< 0.01 , < 0.01
palmitoylcarnitine (C16)	0.17 ± 0.50	0.77 ± 0.43	-0.33 ± 0.64	1.31 ± 0.92	< 0.01 , < 0.01	0.92 , 0.92	< 0.01 , < 0.01
undecenoylcarnitine (C11:1)	0.11 ± 0.62	1.63 ± 0.71	-0.61 ± 0.61†	1.66 ± 0.42	< 0.01 , < 0.01	0.17 , 0.18	< 0.01 , < 0.01
linoleoylcarnitine (C18:2)	-0.09 ± 0.32	0.64 ± 0.38	0.10 ± 0.72	1.66 ± 0.84†	< 0.01 , < 0.01	0.02 , 0.03	< 0.01 , 0.01
dihomo-linolenoylcarnitine (C20:3n3 or 6)	-0.09 ± 0.41	-0.01 ± 0.41	-0.56 ± 0.32	0.02 ± 0.48	0.01 , 0.01	0.13 , 0.14	0.02 , 0.11

**Diacylglycerol**							
diacylglycerol (16:1/18:2 [2], 16:0/18:3 [1])	-0.26 ± 0.63	-0.08 ± 0.61	-2.21 ± 0.68	-1.76 ± 0.63	< 0.01 , < 0.01	0.00 , 0.00	0.07 , 0.21
linoleoyl-arachidonoyl-glycerol (18:2/20:4) [1]	0.11 ± 0.51	0.87 ± 0.53	-0.92 ± 1.09	-0.15 ± 1.17	< 0.01 , < 0.01	0.01 , 0.02	0.92 , 0.94
linoleoyl-arachidonoyl-glycerol (18:2/20:4) [2]	0.02 ± 0.71	0.73 ± 0.65	-1.07 ± 1.11	-0.22 ± 1.11	< 0.01 , < 0.01	0.01 , 0.02	0.45 , 0.71
linoleoyl-docosahexaenoyl-glycerol (18:2/22:6) [1]	0.45 ± 0.83	0.99 ± 0.80	-0.69 ± 1.23	-0.21 ± 1.09	< 0.01 , < 0.01	0.01 , 0.02	0.84 , 0.90
linoleoyl-linolenoyl-glycerol (18:2/18:3) [1]	-0.02 ± 0.90	0.32 ± 0.95	-1.61 ± 1.12	-1.33 ± 1.01	< 0.01 , 0.01	< 0.01 , < 0.01	0.78 , 0.87
linoleoyl-linolenoyl-glycerol (18:2/18:3) [2]	-0.20 ± 1.14	0.33 ± 1.25	-1.10 ± 0.97	-1.31 ± 1.03	0.47 , 0.54	0.01 , 0.01	0.12 , 0.30
linoleoyl-linoleoyl-glycerol (18:2/18:2) [1]	0.20 ± 0.97	0.65 ± 0.94	-1.36 ± 1.47	-0.73 ± 1.44	< 0.01 , < 0.01	0.01 , 0.02	0.32 , 0.57
linoleoyl-linoleoyl-glycerol (18:2/18:2) [2]	0.18 ± 0.96	0.66 ± 1.12	-1.04 ± 1.55	-0.72 ± 1.43	0.01 , 0.01	0.02 , 0.03	0.57 , 0.80
oleoyl-arachidonoyl-glycerol (18:1/20:4) [1]	-0.17 ± 0.72	0.45 ± 0.86	-1.09 ± 0.74	-0.36 ± 0.82	< 0.01 , < 0.01	0.01 , 0.02	0.59 , 0.81
oleoyl-arachidonoyl-glycerol (18:1/20:4) [2]	-0.08 ± 0.71	0.60 ± 0.79	-1.03 ± 0.76	-0.31 ± 0.93	< 0.01 , < 0.01	0.01 , 0.02	0.80 , 0.87
oleoyl-linoleoyl-glycerol (18:1/18:2) [1]	-0.13 ± 1.07	0.24 ± 1.13	-1.63 ± 1.12	-1.16 ± 1.13	< 0.01 , < 0.01	0.01 , 0.01	0.60 , 0.82
oleoyl-linoleoyl-glycerol (18:1/18:2) [2]	-0.12 ± 1.04	0.29 ± 1.09	-1.72 ± 1.07	-1.21 ± 1.10	< 0.01 , < 0.01	< 0.01 , 0.01	0.59 , 0.81
palmitoleoyl-arachidonoyl-glycerol (16:1/20:4) [2]	-0.23 ± 0.69	0.10 ± 0.67	-1.81 ± 0.61	-1.47 ± 0.81	< 0.01 , 0.01	< 0.01 , < 0.01	0.97 , 0.98
palmitoleoyl-linoleoyl-glycerol (16:1/18:2) [1]	-0.26 ± 0.68	-0.02 ± 0.86	-2.04 ± 0.64	-1.85 ± 0.71	0.01 , 0.02	< 0.01 , < 0.01	0.71 , 0.86
palmitoyl-linoleoyl-glycerol (16:0/18:2) [1]	-0.15 ± 1.27	0.39 ± 1.23	-1.72 ± 1.18	-1.17 ± 1.18	< 0.01 , < 0.01	0.01 , 0.01	0.97 , 0.98
palmitoyl-linoleoyl-glycerol (16:0/18:2) [2]	-0.48 ± 0.87	-0.02 ± 0.82	-1.77 ± 1.10	-1.22 ± 1.06	< 0.01 , < 0.01	0.01 , 0.01	0.63 , 0.82
stearoyl-arachidonoyl-glycerol (18:0/20:4) [1]	-0.26 ± 0.76	0.14 ± 0.51	-1.30 ± 0.93	-1.00 ± 0.67	0.01 , 0.01	< 0.01 , < 0.01	0.67 , 0.82

Mean ± SD of log10 transformed change in fatty acid metabolites from BASELINE to PRE and POST aerobic exericse following 24 hours of refeeding to elicit adequate (AD) or low (LOW) glyocgen stores. $n = 11$, *Indicates time-by-treatment interaction, where POST is different than PRE within a treatment; $P < 0.05$, $Q < 0.10$. †Indicates time-by-treatment interaction, where LOW is different than AD at a given time point; $P < 0.05$, $Q < 0.10$
